# L-caldesmon alters cell spreading and adhesion force in RANKL-induced osteoclasts

**DOI:** 10.1186/s12929-019-0505-1

**Published:** 2019-01-24

**Authors:** Chu-Lung Chan, Jiann-Yeu Chen, Ming-Chih Shih, Chih-Lueh Albert Wang, Ying-Ming Liou

**Affiliations:** 1 Department of Life Sciences, National Chung-Hsing University, Taichung, 40227 Taiwan; 2 Research Center for Sustainable Energy and Nanotechnology, National Chung-Hsing University, Taichung, 40227 Taiwan; 3 Department of Physics, National Chung-Hsing University, Taichung, 40227 Taiwan; 4 Boston Biomedical Research Institute, Watertown, MA 02472 USA; 5 The iEGG and Animal Biotechnology Center, and Rong Hsing Research Center for Translational Medicine, National Chung Hsing University, Taichung, 40227 Taiwan

**Keywords:** Osteoclastogenesis, Sealing zone, Non-muscle caldesmon (l-CaD), Cell fusion, TRAP activity, Atomic force microscopy

## Abstract

**Background:**

Osteoclasts (OCs) are motile multinucleated cells derived from differentiation and fusion of hematopoietic progenitors of the monocyte-macrophage lineage that undergo a multistep process called osteoclastogenesis. The biological function of OCs is to resorb bone matrix for controlling bone strength and integrity, which is essential for bone development. The bone resorption function is based on the remodelling of the actin cytoskeleton into an F-actin-rich structure known as the sealing zone for bone anchoring and matrix degradation. Non-muscle caldesmon (l-CaD) is known to participate in the regulation of actin cytoskeletal remodeling, but its function in osteoclastogenesis remains unclear.

**Methods/results:**

In this study, gain and loss of the l-CaD level in RAW264.7 murine macrophages followed by RANKL induction was used as an experimental approach to examine the involvement of l-CaD in the control of cell fusion into multinucleated OCs in osteoclastogenesis. In comparison with controls, l-CaD overexpression significantly increased TRAP activity, actin ring structure and mineral substrate resorption in RANKL-induced cells. In contrast, gene silencing against l-CaD decreased the potential for RANKL-induced osteoclastogenesis and mineral substrate resorption. In addition, OC precursor cells with l-CaD overexpression and gene silencing followed by RANKL induction caused 13% increase and 24% decrease, respectively, in cell fusion index. To further understand the mechanistic action of l-CaD in the modulation of OC fusion, atomic force microscopy was used to resolve the mechanical changes of cell spreading and adhesion force in RANKL-induced cells with and without l-CaD overexpression or gene silencing.

**Conclusions:**

l-CaD plays a key role in the regulation of actin cytoskeletal remodeling for the formation of actin ring structure at the cell periphery, which may in turn alter the mechanical property of cell-spreading and cell surface adhesion force, thereby facilitating cell-cell fusion into multinucleated OCs during osteoclastogenesis.

**Electronic supplementary material:**

The online version of this article (10.1186/s12929-019-0505-1) contains supplementary material, which is available to authorized users.

## Background

Osteoclasts (OCs) are multinucleated, bone-resorbing cells that differentiate from hematopoietic progenitors of the monocyte-macrophage lineage through a multistep process called osteoclastogenesis, including cell commitment, cell-cell fusion, and maturation [1, 2]. The key event for the regulation of OC differentiation is the RANKL-RANK-TRAF signalling pathway that leads to the activation of MAPK, NF-κB, and c-Src for upregulation of the transcriptional factors, i.e., AP-1 and cNFATc1, and turning on the expression of osteoclast marker genes, i.e., cathepsin K, TRAP, and β3 integrin [3]. The activation for c-Src and the downstream kinases also induces the formation of the actin-rich structures known as podosomes [4]. Podosomes are specialized actin-rich membrane protrusions and serve as sites for cell adhesion or extracellular matrix degradation [5, 6]. In the process of the OC differentiation and maturation, the assembly of podosome starts as clusters of actin bundles, and develops into small rings, and finally encircles the cell periphery in a podosome belt as OCs become mature. When an OC is attached to the bone surface, a sealing zone is formed for bone resorption [7]. Because OCs are key players for bone resorption, they are one of the main targets for treatment of osteoporosis based on the critical events of podosome formation in osteoclastogenesis [8, 9].

Caldesmon (CaD), which exists in two isoforms, h-CaD (the high molecular weight form) and l-CaD (the low molecular weight form) in smooth muscle and non-muscle cells, respectively, is capable of stabilizing actin filaments, inhibiting actomyosin ATPase activity, and protecting actin filaments against severing proteins (e.g., gelsolin) [10, 11]. There are two serine/threonine-containing regions in the C-terminal domain of both h- and l-CaD that may be phosphorylated by a number of upstream kinases, including PKC, CAM Kinase II, cdc2 kinase and Erk1/2 MAPKs [18–23]. Phosphorylation by either of these kinases reverses the inhibitory effects of CaD. It is likely that phosphorylation results in weakened CaD binding to actin and allows severing proteins to disassemble the actin cytoskeleton, hence freeing CaD to move to the cell periphery where the cytoskeleton undergoes reassembling [14, 15]. Although CaD, as well as other actin binding proteins (ABPs) are known to participate in the dynamic assembly of the podosome in several types of invasive and motile cells [12–14], little is known about the role of l-CaD in the control of the podosome formation and cell-cell fusion in osteoclastogenesis.

Recently, we have shown that the expression of l-CaD in response to RANKL activation is an important event for osteoclastogenesis [15]. In addition, our study using decoy peptides as well as full-length mutants mimicking the phosphorylation of l-CaD suggested that l-CaD phosphorylation plays a role in the control of the F-actin architecture of podosome and the substrate resorption function in differentiated OCs upon RNAKL activation [15]. Here, we further determined the role of l-CaD in the regulation of OC fusion in osteoclastogenesis by using the approach of gain and loss of the l-CaD level in RAW264.7 cells followed by RANKL induction. Atomic force microscopy (AFM) was used to assess the mechanical properties such as spreading and surface adhesion force of RANKL-induced OCs with and without l-CaD overexpression or si l-CaD. Our data indicated that l-CaD participates in organizing the actin ring structure at the cell periphery of differentiated OCs, which may in turn facilitate the cell-cell fusion into mature OCs by altering their cell-spreading and adhesion force during osteoclastogenesis.

## Methods

### Chemicals and reagents

RAW264.7 cell lines were purchased from American Type Culture Collection (ATCC, CRL-1446) (Rockville, MD). All reagents used were ACS or MB grade. sRANKL and mCSF recombinant mouse proteins were purchased from R&D Systems (Minneapolis, MN).

### Cell culture and RANKL treatment

RAW264.7 cells in regular Dulbecco’s modified essential medium (DMEM) (Gibco, Thermo Fisher Scientific Inc., Waltham, MA) supplemented with antibiotics and 10% fetal bovine serum (FBS) were cultured in a 5% CO_2_ incubator at 37 °C. For differentiation, cells were cultured in minimal essential medium alpha modification (α-MEM, Gibco) with 50 ng/ml RANKL with or without 20 ng/ml mCSF for 5 or 6 days, changing with the fresh RANKL-containing differentiation medium at day 3. OC differentiation was assessed by tartrate resistant alkaline phosphatase (TRAP) activity using the Leukocyte Acid Phosphatase kit (Sigma-Aldrich, St. Louis, MO). Cells were fixed and stained for TRAP activity according to the manufacturer’s instructions.

### Immunoblotting

Protein contents of total cell lysates from RANKL treated or untreated cells were analyzed by western blot. Samples with same amounts of protein were separated by sodium dodecyl sulphate-polyacrylamide gel electrophoresis, then the proteins were electro-transferred onto polyvinylidene difluoride membranes. The primary antibodies used were: rabbit polyclonal anti-caldesmon (Sigma SAB4503189; 1:500 dilution), rabbit polyclonal anti-p-caldesmon (Ser789) (Santa Cruz Biotechnology, Dallas. TX, sc-12,931-R, 1:500), mouse monoclonal anti-human vinculin (7F9) (Santa Cruz sc-73,614, 1:1000 dilution), mouse monoclonal anti-β-actin (Sigma, 1: 10000 dilution), and rabbit polyclonal anti-GAPDH (Sigma SAB4300645; 1:5000). The secondary antibodies used (1:5000 dilution) were goat anti-rabbit IgG (Sigma), and goat anti-mouse IgG (Sigma).

### Resorption pit formation assay

The resorption pit formation assay was described previously [15]. In brief, RAW264.7 cells (5000 cells per well) were transferred into each well of a Corning Osteo Assay Stripwell plate (Corning Cat. No.3989) to begin the differentiation process. Plates were incubated at 37 °C in a humidified atmosphere of 5% CO_2_ for 7 days with a medium change on day 3 or 4. To analyse the surface for pit formation, the media were aspirated from the wells on day 7, and 100 μL of 10% bleach solution (HOCl) was added for 5 min at room temperature. The wells were washed twice with distilled water and allowed to dry at room temperature for 3 to 5 h. Individual pits or multiple pit clusters were observed using a microscope at 100x magnification. The images containing the pit area were analysed using Image J software version 1.50a (NIH, USA).

### Immunocytochemistry, fluorescence staining, and laser-scanning confocal microscopy

RAW264.7 cells (2*10^4^) were seeded on a glass coverslip in each well of a 12-well plate and cultured in DMEM-10% FBS for 24 h in a CO_2_ incubator at 37 °C; after this time period, the medium was added with 50 ng/ml RANKL. On days 5 or 6, the cells were fixed in 4% paraformaldehyde for 15 min, permeabilised with 0.1% Triton X-100 for 1 min, blocked with 2% bovine serum albumin for 30 min (all at room temperature), and then incubated with primary antibodies in phosphate buffer saline (PBS). They were then incubated for 30 min at room temperature with secondary antibody (1: 700 in PBS) conjugated to a fluorescence probe (Alexa Fluor® 488), then F-actin was labelled by incubation for 5 min at room temperature with Alexa Fluor® 568 phalloidin (1: 1000) (Molecular Probes, Invitrogen Life Sciences; Carlsbad, CA) and the nucleus labelled with DAPI (1: 1000) (Molecular Probes) for 1 min. Immunofluorescence images were captured on a Leica TCS MP5 confocal microscope controlled by the manufacturer’s Leica Confocal Software package LAS AF version 2.63 using a 63 × 1.4 Plan Apochromatic oil-immersion objective (Leica Microsystems, Exton, PA). Different optical sections from the bottom to the top with an increase of 0.1 μm per section were obtained for image analysis. The raw fluorescence images without further processing were used for data analysis. Fluorescence intensity measurements and three-dimensional image reconstructions were performed in Image J software version 1.50a (NIH, USA).

### Atomic force microscopy

For atomic force microscope (AFM) experiments, cells were seeded in a density of 10,000 cells on 20-mm round coverslips immersed into 12 well culture dishes and cultured in 10% FBS containing α-MEM (Gibco) with 50 ng/ml RANKL for 5 days, changing differentiation medium once at day 3. Before measurement, the medium was exchanged against PBS.

The AFM System-Bruker Dimension Icon with ScanAsyst® (Bruker Corporation, Billerica, MA) was used to resolve nanoscale surface topography and to quantitatively map the mechanical property of the cell surface by scanning a sharp probe over the cell surface in phosphate buffer. The spring constant of the cantilever was determined before the measurement. For determining cell spreading, the height sensor images obtained with cell surface topography of height scanned in each optical field containing the cell of interest and background were used to obtain the height depth histogram. Height sensor signal was used to display the cell surface image using Nanoscope Analysis v1.40 (Bruker Nano Surfaces, Santa Barbara, CA). After threshold of background, the minimized depth (height) was set as the depth of the cell boundary. Then, the normalized bearing area vs the relative depth surrounding the periphery to the center of the cells was plot. The percentage of bearing area for the peripheral area to the whole cell area was used to estimate the tendency of cell spreading in this study. For measuring the adhesion force exerting on the cell surface, the mode of peak force capture was used to analyze 10 individual force-distance curves at discrete points randomly selected from the region outside the cell, at the cell periphery, and the region inside the cell, respectively. For each single curve captured, the backward force-distance curve was used to determine the adhesion force at each point. The captured points outside the cell were used as the background measurements.

### Statistical analysis

Quantitative values are presented as the mean and standard error of the mean (mean ± SEM). A difference was considered to be statistically significant when the *P* value was less than 0.05.

## Results

### L-CaD is associated with the formation of actin ring in RANKL-induced osteoclastogenesis

**Fig. 1:**
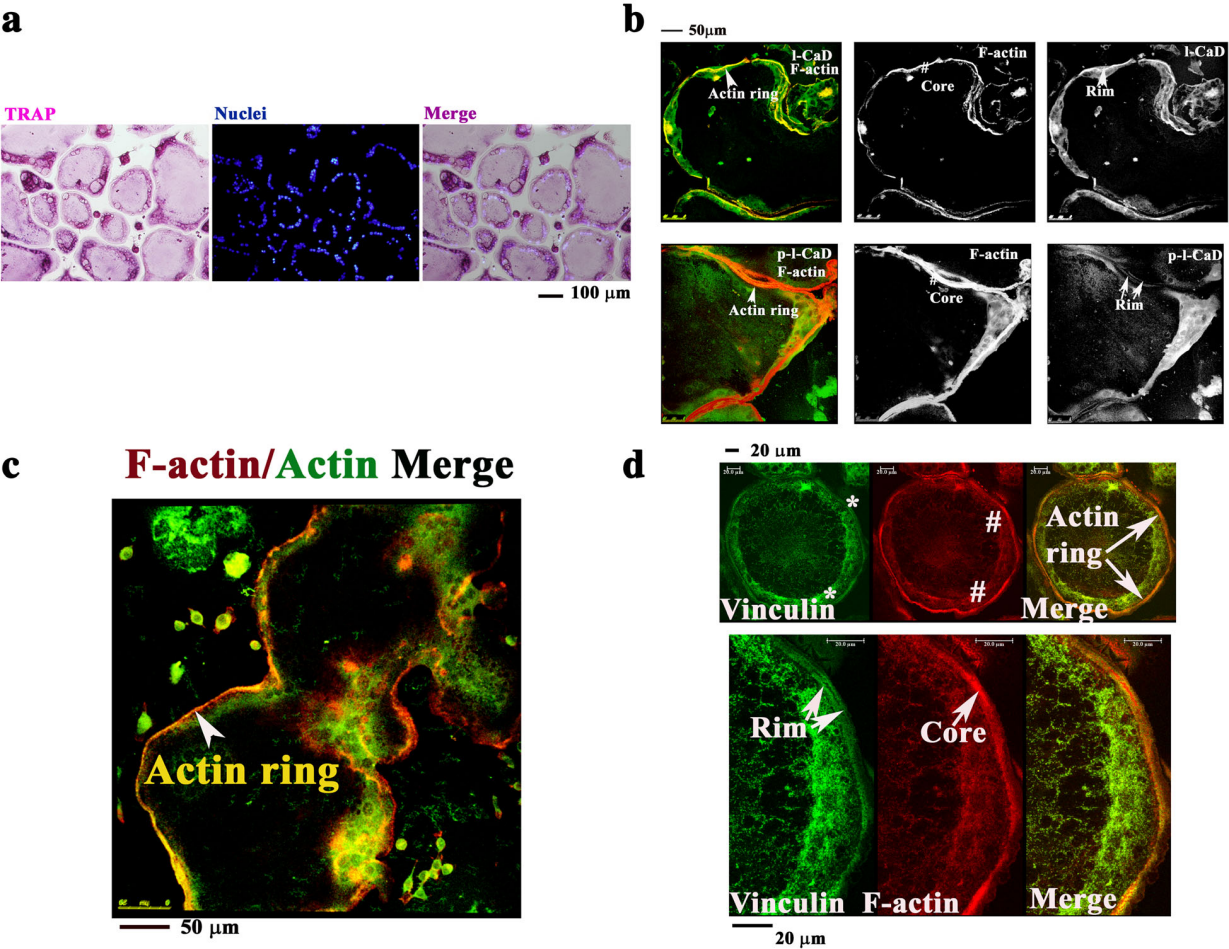
RANKL-induced differentiation of RAW264.7 cells. **a** Characteristic TRAP-stained RAW264.7 cells with RANKL induction for 5 days. Multinucleated OCs were observed by TRAP and nuclei staining with DAPI. **b** OCs characterized with actin ring formation around the cell periphery by using F-actin fluorescent staining with rhodamine phalloidin (red) and immuno-fluorescent staining β-actin (green). **c** Actin ring structure showing the core as indicated by # in RANKL-induced OC cells stained with l-CaD (right top) and phosphorylated l-CaD (p-l-CaD; right bottom), F-actin (middle), and merged color micrograph showing l-CaD staining (left top) and p-l-CaD (left bottom) in green, F-actin in red, and colocalized stains in yellow. Calibration bars in (**a**), (**b**), and (**c**) as indicated, respectively. **d** Actin ring structure composed of the core as indicated by # (labelled with F-actin as red in the top middle panel) and the peripheral rim as indicated by * (labelled with vinculin as green in the top left panel) and merged color micrograph (the top right panel) showing the actin ring as indicated by white arrow. Magnified portion (right bottom) showing the actin ring structure with the peripheral rim labelled with vinculin around the core in the center with red F-actin staining. Calibration bar: 20 μm as indicated in each panel

During RANKL-induced differentiation, RAW264.7 cells undergo characteristic changes of increased cell-cell fusion into large and multinucleated TRAP-positive OCs (Fig. 1a). In addition, RANKL activation also causes the formation of an actin ring around the cell periphery in OCs (Fig. 1b). The actin ring structure is composed of two major domains: a central core that involves dynamic polymerization and depolymerization of actin filaments and an adhesion ring domain that contains cell-matrix focal adhesions [6]. Previously, we have shown that l-CaD is associated with the actin core structure in the RANKL-induced actin ring in osteoclastogenesis [15]. Consistently, l-CaD was found to co-localize with the F-actin within the actin core while move to the cell peripheral as being phosphorylated (Fig. 1c), where vinculin, a membrane-cytoskeletal protein contributed to the linkage of integrin adhesion molecules to the actin cytoskeleton [5], was also found to reside at the rims of the actin core in differentiated OCs (Fig. 1d).

### L-CaD expression levels modified the actin ring structures in OCs and their mineralized matrix degradation

**Fig. 2:**
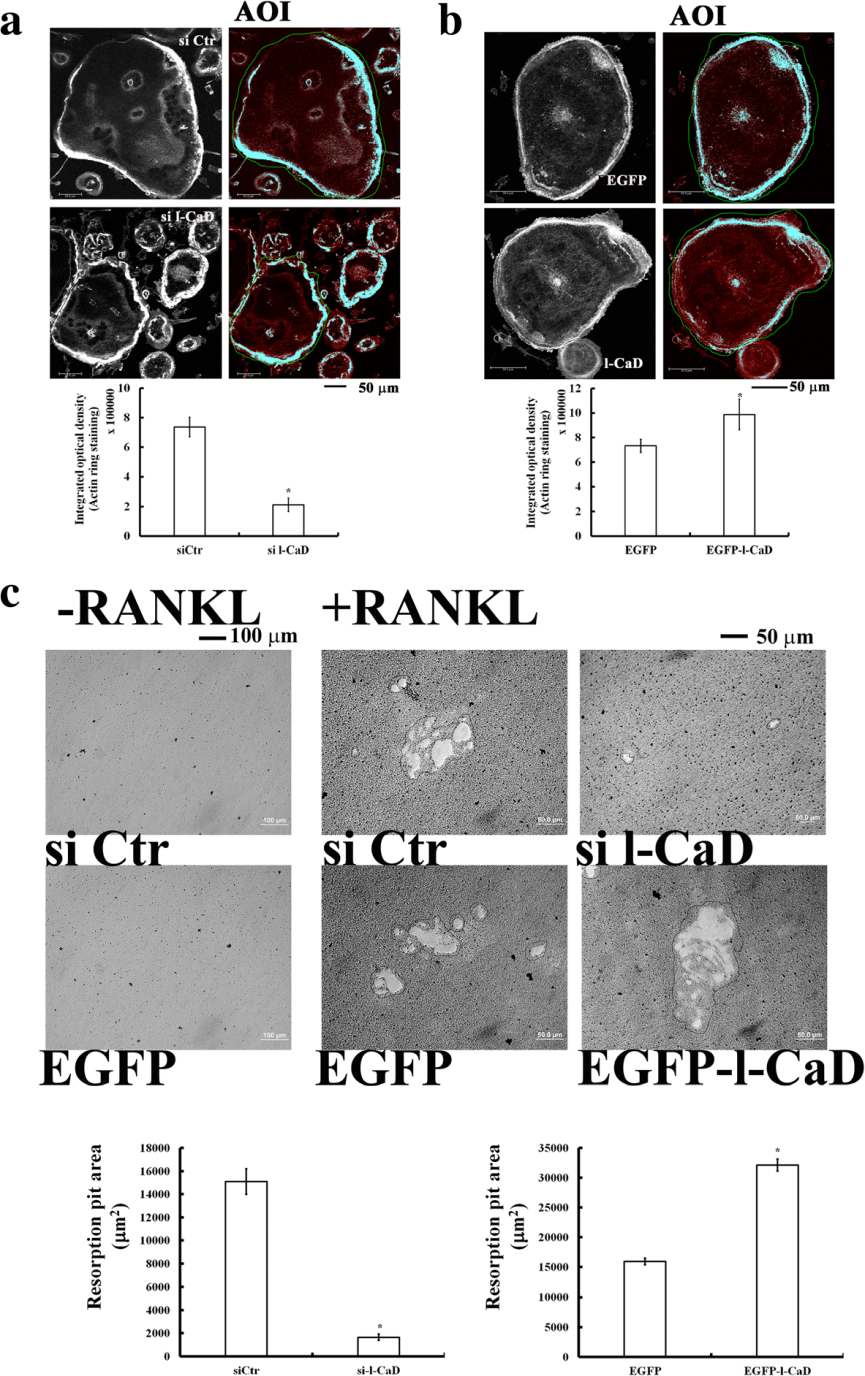
l-CaD expression levels alter the actin ring structures in OCs associated with their mineralized matrix degradation. **a** Actin ring formation labeled with rhodamine phalloidin in RANKL-induced OCs treated with control siRNA (top, siCtr) and si-l-CaD (bottom). The area of interest (AOI) for the actin ring (highlighted in indigo blue) in OCs (delimited by a line of green circle) as shown on the left was measured by using Image-Pro Plus computerized software. Quantitative analyses of the optical density for the actin ring forming area in each cell with si Ctr and si-l-CaD are shown at the bottom. **b** Actin ring formation in RANKL-induced OCs transfected with EGFP controls (EGFP, top) or EGFP-l-CaD (l-CaD, bottom). Quantitative analyses of the optical density for the actin ring forming area in each cell transfected with EGFP and EGFP-l-CaD are shown at the bottom. **c** Resorption pit assay showing the l-CaD dependent changes of resorption pit forming area in RANKL-induced OCs treated with si Ctr or si-l-CaD, or transfected with EGFP control and EGFP-l-CaD vectors. Resorption pit assay in cells without RANKL induction was shown on the left column. Calibration scale as indicated. In **a**, **b**, and **c** the values are the mean ± SEM (*n* = 6), with * indicating a significant difference compared to the si Ctr-treated or EGFP-transfected cells, respectively

To determine the role of l-CaD in forming actin ring structures in OCs, gain- and loss-of-functions in RAW264.7 cells overexpressing l-CaD (Supplementary S2) and treated with si l-CaD (Additional file 1: Figure S1), respectively, followed by RANKL induction were used. The actin ring formation in differentiated OCs was visualized by using a confocal microscope along with fluorescent staining of F-actin with rhodamine phalloidin (Figs. 2a and b). In comparison with the si Control (si Ctr), si l-CaD knock-down cells became smaller in size and decreased 71% of the actin ring staining (Fig. 2a). In contrast, overexpression of l-CaD in cells transfected with a fusion DNA construct that contained EGFP and l-CaD (Additional file 2: Figure S2) had 1 to 2-fold increases of actin ring staining at the cell periphery relative to the EGFP-transfected control cells (Fig. 2b). In addition, bone resorption assays (Fig. 2c) indicated that gene silencing of l-CaD (with si l-CaD) had a 90% decrease in the formation of bone resorption pit area as compared to siCtr. When compared to the EGFP-transfected control, l-CaD overexpression showed 2.3-fold increases in the formation of bone resorption pit area (Fig. 2c). Clearly, gene silencing of l-CaD attenuates the formation of actin ring in concomitant with bone resorption, while l-CaD overexpression facilitates the actin ring formation and resorption activities in RANKL-induced OC cells.

### Effect of l-CaD overexpression on the localization of l-CaD/phosphorylated l-CaD to the F-actin associated with the actin ring structure in differentiated OCs

**Fig. 3:**
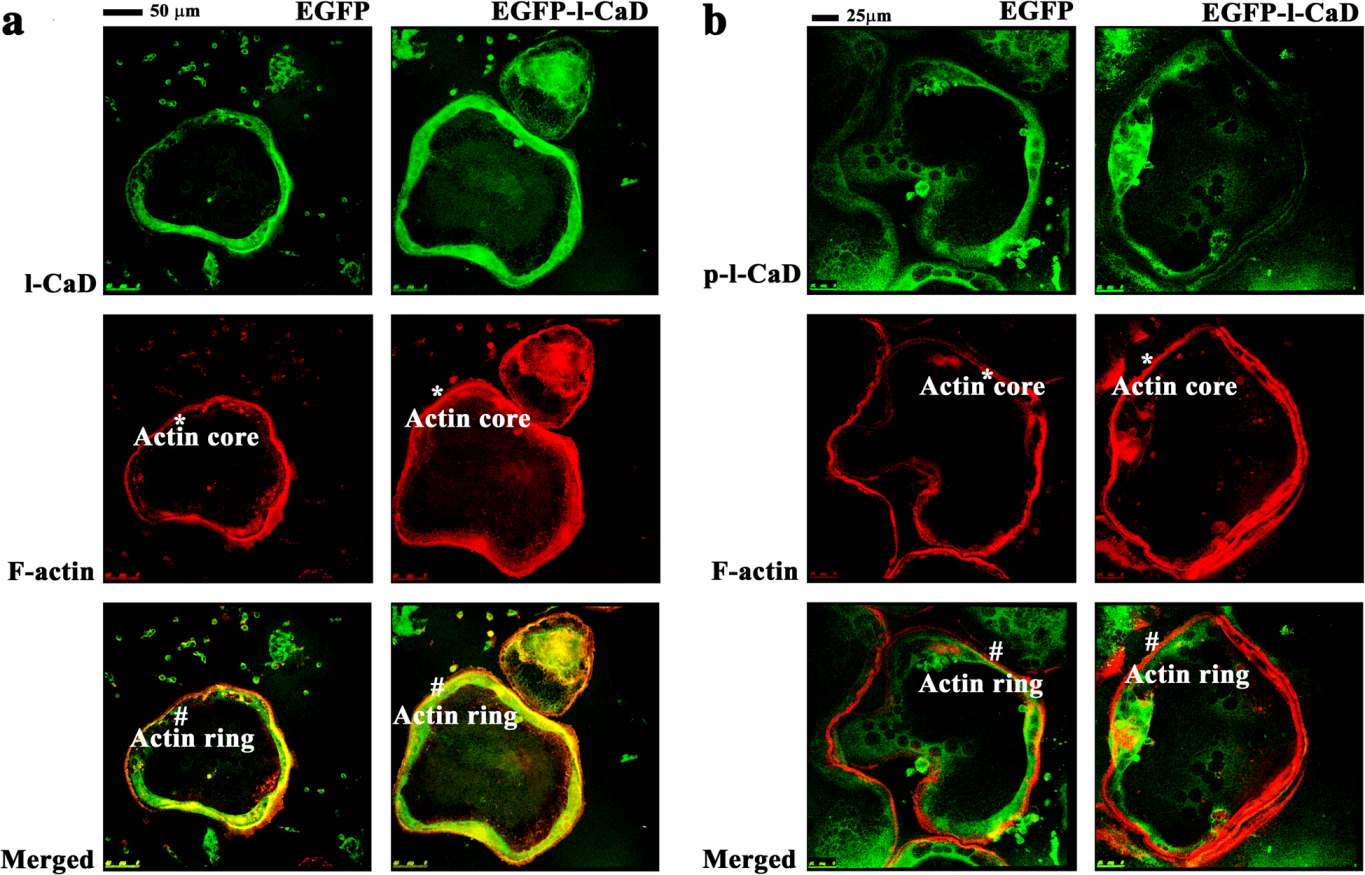
Effect of l-CaD overexpression on the colocalization of l-CaD/phosphorylated l-CaD to the F-actin associated with the actin ring structure in differentiated OCs. **a** Laser scanning confocal microscopy showing the images of RANKL-induced actin ring structures stained with l-CaD (green, top) and with F-actin (red, middle) in OCs transfected with EGFP and EGFP-l-CaD vectors, respectively. The actin core as indicated by * showing the F-actin rich staining with rhodamine phalloidin (red) at the periphery of the cells and actin ring as indicated by # showing the colocalized portion with the yellow core in the center. Calibration bar 50 μm as indicated. **b** The stacking confocal micrographs showing phosphorylated l-CaD staining in green (top), F-actin staining in red (middle), and merged images in orange or yellow (bottom). The actin ring and actin core as indicated showing the colocalized portion with the phosphorylated l-CaD and F-actin in the periphery. Calibration bar 50 μm as indicated

To show if the augmented actin ring formation is associated with the increased l-CaD level in the actin ring structure of differentiated OCs, RANKL-induced precursor cells transfected with EGFP-l-CaD and EGFP control vectors were studied for their localization of l-CaD/phosphorylated l-CaD to the F-actin associated with the actin ring structures (Fig. 3). Here, we applied primary antibodies that recognize both unphosphorylated and phosphorylated l-CaD (endogenous and exogenous) in transfected cells, followed by secondary antibody staining. For phosphorylated l-CaD detection, we used another antibody that specifically recognizes phosphorylated (at the Erk-sites) form of l-CaD. F-actin was stained by rhodamine phalloidin. Our results showed that the RANKL-induced cells with EGFP-l-CaD transfection had significantly increased the rhodamine phalloidin staining at the F-actin-rich core in concomitant with the total l-CaD staining (Fig. 3a). However, the increased F-actin associated with the actin core did not co-localize with phosphorylated l-CaD in OCs, since the latter became dissociated from the actin core in the RANKL-induced cells with (Fig. 3b) or without l-CaD overexpression (Fig. 1c). This is consistent with our reported finding that the majority of the increased l-CaD or overexpression in RANKL-induced cells remained unphosphorylated and was localized to the actin-rich core of the actin ring, but moved to the peripheral as being phosphorylated [15].

### L-CaD facilitates cell-cell fusion in RANKL-induced OCs

**Fig. 4:**
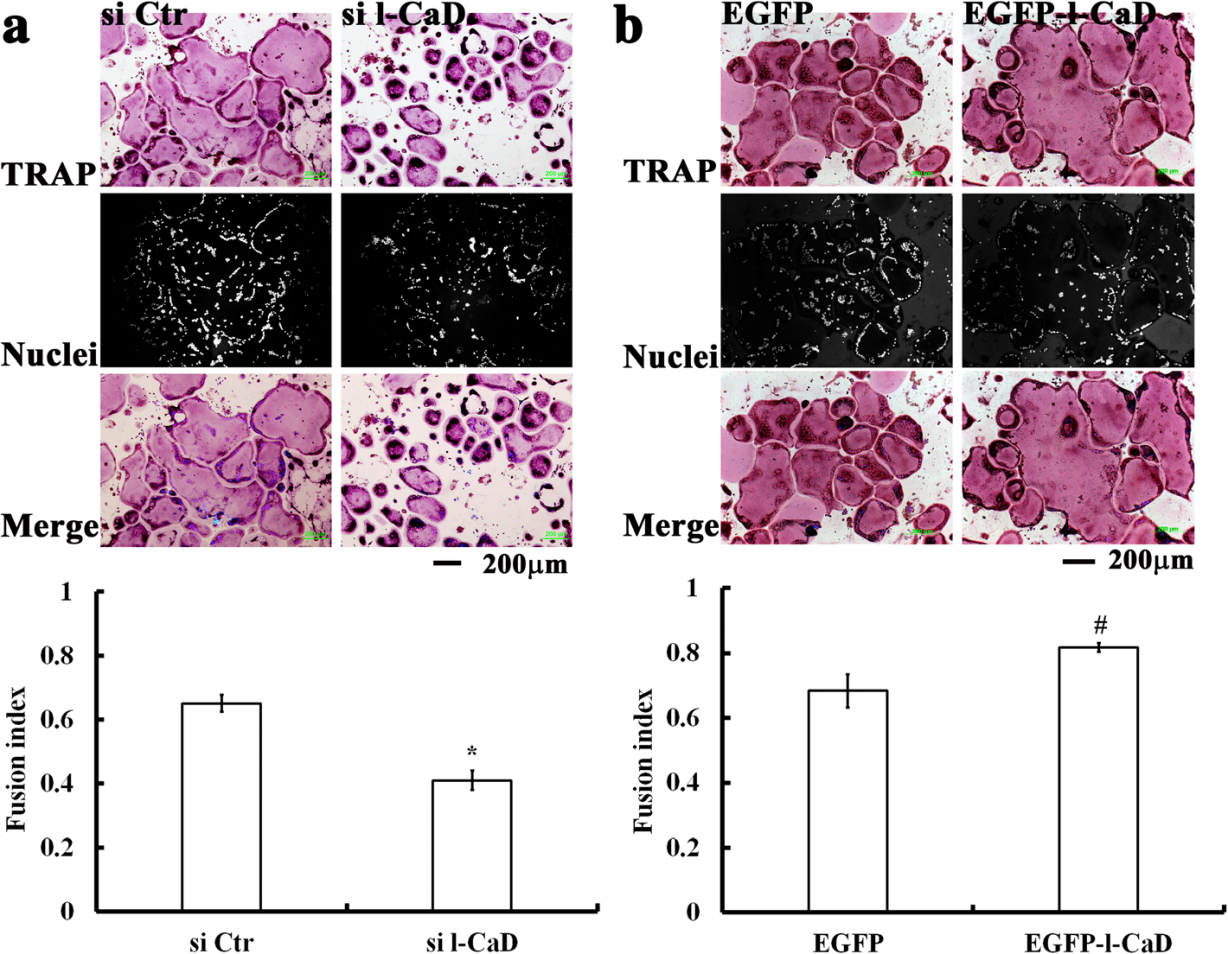
l-CaD facilitates the cell-cell fusion in RANKL-induced OCs. (**a**) RANKL-induced TRAP-positive OC images with TRAP staining (red, top), and DAPI nuclei staining (white, middle), and merged color micrographs showing TRAP staining in red, and nuclei in blue in differentiated cells with si Ctr or si-l-CaD treatment. Fusion index was determined by measuring the portion of clustered nuclei to total nuclei in cells by using Image-Pro Plus computerized software. Quantitative analyses for RANKL-induced cell fusion index in OCs treated with si Ctr or si-l-CaD were shown at the bottom. **b** RANKL-induced TRAP-positive OC images with TRAP staining (red, top), and DAPI nuclei staining (white, middle), and merged color micrographs showing TRAP staining in red, and nuclei in blue in OCs with EGFP or EGFP-l-CaD transfection. Quantitative analyses for RANKL-induced cell fusion index in OCs transfected with EGFP or EGFP-l-CaD expressions were shown at the bottom. Calibration scale in (**a**) and (**b**) as indicated. In (**a**) and **(b),** the values are the mean ± SEM (*n* = 6), with *, # indicating a significant difference compared to the si Ctr-treated or EGFP-transfected cells, respectively

It has been shown that cell-cell fusion of precursor cells into multinucleated OCs enhanced their efficiency of bone resorption by forming actin rings at cell peripheries [5–7]. To determine the role of l-CaD in the multinucleation of OCs differentiated by RANKL induction, a cell fusion index [15, 17] was calculated by the portion of clustered nuclei (measured by the staining of nuclei with DAPI) in the TRAP-containing regions relative to the total nuclei in RANKL-induced cells with and without l-CaD overexpression (Fig. 4b) or gene silencing (Fig. 4a). In comparison to that of the si Ctr, gene silencing of l-CaD decreased the number of larger OC cells with a diameter greater than 300 μm, whereas the number of smaller OC cells (diameters less than 150 μm) increased (Fig. 4a). This change in cell size accounts for 37.5% decrease of cell fusion index for the cells with si l-CaD (Fusion Index = 0.40) compared to the control cells (Fusion Index = 0.64) (Fig. 4a). On the other hand, l-CaD overexpression promoted OC cell differentiation with an increased number of larger OC cells (diameters greater than 450 μm), but decreases the number of smaller OC cells (diameters less than 300 μm), as compared to controls (Fig. 4b); this change resulted in a higher degree of cell-cell fusion in l-CaD overexpressing cells (Fusion Index ~ 0.82), as compared to EGFP control cells (Fusion Index ~ 69%) (Fig. 4b). This observation supports the hypothesis that elevated l-CaD expression facilitates cell-cell fusion in OC cell differentiation [15].

### Effects of l-CaD on mechanical changes of cell spreading and adhesion force in RANKL-induced cells

**Fig. 5:**
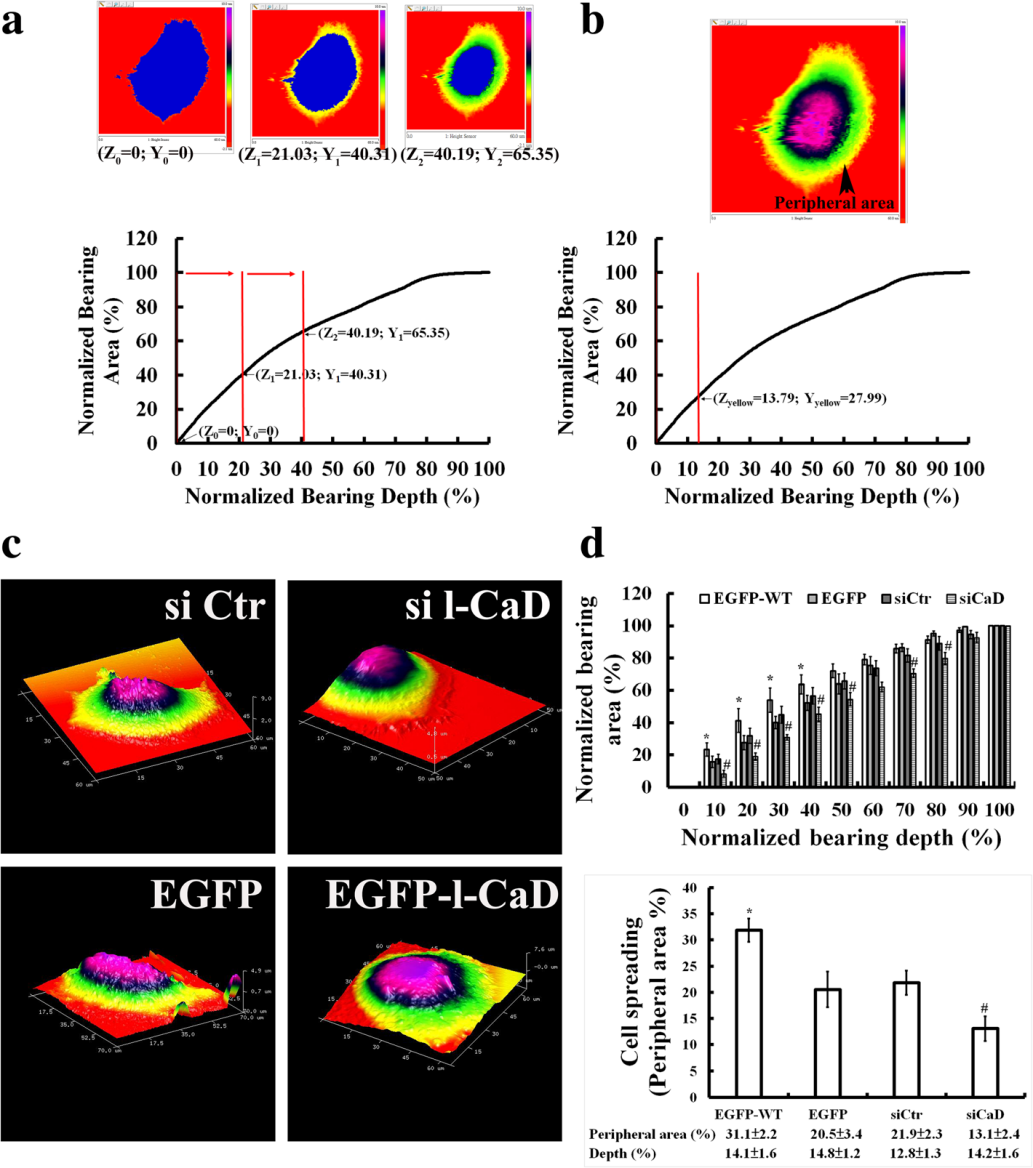
Effects of l-CaD on mechanical changes of cell spreading in RANKL-induced cells. **a** The height sensor image obtained with AFM scanning cell surface topography of height. Using the bearing analysis (built-in software of NanoScope Analysis v1.40), bearing area (Z) versus the bearing height (X) was adjusted to find the threshold for obtaining the full masking with blue on the whole cell (left panel). The threshold was used to define the minimized cell height at the cell boundary (X_0_, Z_0_), and set the maximal area bearing within the cell, and then normalize the relation curve describing the bearing area (%) to the bearing height (%) for the cell of interest. With increasing the height from 20 to 40% accompanied by increasing the bearing area as indicated from the cell periphery (middle panel) toward the center (right panel) of the cell. **b** Pseudo-coloring the cell of interest was used to find the peripheral area of the cell in the yellow coloring zone, with the percentage of bearing area (Z_yellow_) and cell height (X_yellow_) as indicated. The bearing area covering the cell peripheral region (yellow zone) was used to estimate the tendency of cell spreading for the cells. **c** The 3D presentation for the height sensor image obtained with AFM scanning cell surface topography of height in RANKL-induced cells treated with si Ctr or si-l-CaD, or transfected with EGFP control or EGFP-l-CaD. **d** Quantitative analyses for the l-CaD dependent changes of the relation curve of normalizing bearing area (Z_yellow_) and cell height (X_yellow_) (top), and the cell spreading (bottom) in cells treated with si Ctr or si-l-CaD, or transfected with EGFP control and EGFP-l-CaD. The values are the mean ± SEM (*n* = 6), with *, # indicating a significant difference compared to the si Ctr-treated or EGFP-transfected cells, respectively

**Fig. 6:**
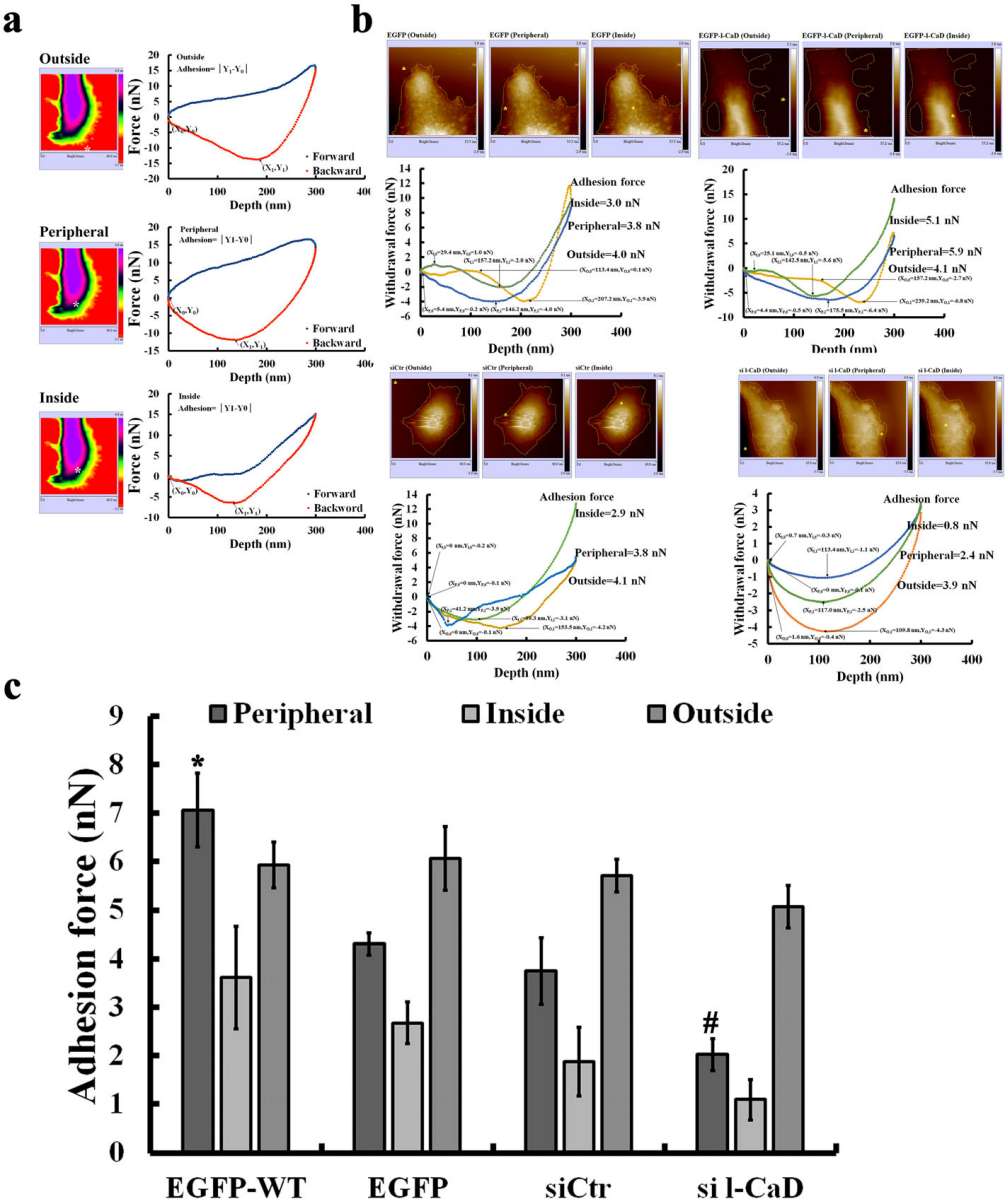
Effects of l-CaD on mechanical changes of adhesion force in RANKL-induced cells. (**a**) After AFM imaging the mechanical property of the cell surface, the recorded image at the adhesion mode was analyzed for the force curves at pixels of interest (i.e. the region outside the cell, at the cell periphery, and the region inside the cell), as indicated by *. The adhesion force at outside was used as the background adhesion force measurement. (**b**) The backward force vs tapping depth curves were used to calculate the adhesion force for cells transfected with EGFP or EGFP-l-CaD, or treated with si Ctr or si-l-CaD. (**c**) Quantitation of the l-CaD effect on the adhesion force at the region outside the cell, at the cell periphery, and the region inside the RANKL-induced cells with EGFP or EGFP-l-CaD(EGFP-WT), or with siCtr or si-l-CaD. The values are the mean ± SEM (n = 6), with *, # indicating a significant difference compared to the si Ctr-treated or EGFP-transfected cells, respectively

Precursor cell fusion into multinucleated OCs involves cell migration, repeated attachment and detachment of cells to the substrate, aggregation, spreading to the matrix, and intercellular fusion of plasma membranes [1, 2, 16]. To determine whether l-CaD is involved in modulating OC cell differentiation by modifying cell surface mechanics in osteoclastogenesis, AFM was used to probe mechanical changes of cell spreading (Fig. 5) and cell surface adhesion force (Fig. 6) in RANKL-induced OC cells with and without l-CaD overexpression or gene silencing. Cell spreading was measured by the height scanning mode of AFM (Fig. 5) to obtain the percentage of the bearing area at different cell depths (Fig. 5a). The bearing area covering the cell peripheral region with the less cell depth was used to estimate the tendency of cell spreading for the cells with and without l-CaD overexpression or gene silencing (Fig. 5b,c). Our data showed that overexpression of l-CaD increased cell spreading by 10%, whereas gene silencing of l-CaD decreased cell spreading by 8% (Fig. 5d).

After AFM imaging the mechanical property of the cell surface, the recorded image at the adhesion mode was analyzed for the force curves at pixels of interest (i.e., outside the cell, at the cell periphery, and inside the cell; Fig. 6a). The adhesion force was calculated by using the backward force-depth curve for the cells with and without l-CaD overexpression or gene silencing (Fig. 6b). The scanning area outside the cell was used as the background measurement. Our data showed that l-CaD overexpression increased adhesion force at the cell periphery by 3 nN, while gene silencing of l-CaD decreased the adhesion force at the cell periphery by 2 nN. However, no significant difference of the adhesion force inside the cell was found among cells with and without l-CaD overexpression or gene silencing (Fig. 6c).

## Discussion

In this study, we showed that l-CaD is associated with the formation of actin ring during RANKL-induced osteoclastogenesis (Fig. 1). The expression level of l-CaD was found to affect the ability of the OCs to form actin ring (Fig. 2a and b), to degrade mineralized substrates (Fig. 2c), and to fuse into multinucleated cells (Fig. 4). Overexpressed l-CaD seemed to remain largely unphosphorylated and to localize to the actin-enriched core of actin rings, but translocate to the peripheral as being phosphorylated (Fig. 3). In addition, with the use of AFM to resolve the mechanical changes of cell surface topography, we provided evidence to show that gain or loss of l-CaD expression level alters cell spreading (Fig. 5) and the adhesion force (Fig. 6) in RANKL-induced OCs. Our study suggested that l-CaD-dependent remodeling of the actin cytoskeleton modulates mechanical properties of cell surface (i.e., cell spreading and adhesion force) and alters OC fusion and matrix degradation.

It becomes apparent that the actin cytoskeleton is important for OCs, not only in terms of structural support, but also for cell adhesion, polarization, and migration [17]. During the process of maturation, OCs first form a sealing zone, an actin-rich ring structure resembling densely packed podosomes, at the ventral surface of cells [6, 7]. Inside the sealing zone, proton pumps and chloride channels export these ions to acidify the surface in contact with the ruffled border. As a consequence, the mineral component of bone matrix is dissolved, allowing phosphatases (i.e. TRAP) and proteolytic enzymes (i.e. cathepsin K) to degrade mineralized bone matrix, therefore creating a resorption lacuna. To accomplish their function, OCs repeat between resorption and migration along the bone surface, requiring dynamic actin cytoskeleton remodelling [6, 7].

Podosomes are dynamic actin-rich protrusive structures involving polymerization/depolymerization of the central F-actin core surrounded by an adhesion ring, containing integrin, cytoskeletal adaptor proteins, and actin network filaments [18–20]. Consistent with our recent report [15], this study showed colocalization of l-CaD with F-actin within the actin core, while phospho-l-CaD dissociated from the core (Fig. 1c) and vinculin organizing the adhesion ring appeared at the rims of the actin core (Fig. 1d) in OCs [5]. Regulators of podosome include tyrosine kinases [21], RhoGTPases [22], actin regulators [23] and the microtubule system [24]. During OC maturation, activation of α_V_β_3_ integrin stimulates c-Src and induces phosphorylation of Pyk2 and p130Cas, along with vinculin and paxillin to organize the adhesion complex formation, and that establishes a link with the actin filament network, known as the adhesion ring [25]. This adhesion ring is then connected to the F-actin core via membrane-bound cdc42 [26] to activate the formation of cortactin/WASP-Arp2/3 complex for actin polymerization [27]. Alternately, another route by the PI3K-induced modulation of phosphoinositol/Ca^2+^ signalling cascades initiated from the adhesion ring could also modify the activity for actin depolymerisation by gelsolin which occurs at the actin core [28]. Apparently, there is a dynamic connection between the adhesion ring and the F-actin core for the podosome formation in OCs and their substrate degradations. In this study, our experiments using gain- and loss-of-function showed that the expression levels of l-CaD could modify the formation of actin ring (densely packed podosomes) in OCs (Fig. 2a and b) with concomitant changes in their bone resorbing function (Fig. 2c). It appears that l-CaD acts to modify the dynamic assembly of the podosome structures in OCs and their bone-resorbing function.

A study with dendritic cells provided evidence for that a structural link established between protrusive cores and adhesion rings at the podosomes could be coordinated by the interplay between myosin IIA-mediated contractility and actin network integrity [29]. In that study, the podosome core was found to grow with actin polymerization and generate tension within the F-actin core [29]. Then, the tension sensed by the adhesion ring could recruit vinculin and zyxin to stabilize the podosome integrity. On the other hand, myosin IIA could also contract the actin filament networks and exert tension on vinculin, overcoming core growth and eventually attenuating podosome size and protrusion [29]. Based on these findings, the authors suggested that dendritic cells could use this modeled podosome structure in coordination with the actomyosin apparatus to sense and remodel extracellular matrix environmental cues [29, 30]. In addition, another study also identified the unconventional single-headed myosin IXB that contains an RhoGAP domain at its tail [31, 32] exerting its ability to modulate the podosome patterning and OC function [33]. These findings raised the possibility that contractile interaction between actin and non-muscle myosin regulated by the actin filament-stabilizing proteins could modulate the dynamic assembly of the podosome structure. CaD is known to regulate the actin-myosin interaction in both smooth muscle and non-muscle cells by the mode of phosphorylation/dephosphorylation [34, 35]. l-CaD has been shown to localize to the actin-enriched core of podosomes, but move to the adhesion ring once phosphorylated [14, 15]. In this study, we also showed that l-CaD overexpression in differentiated OCs has significantly increased the rhodamine phalloidin staining at the F-actin-rich core in concomitant with the increased l-CaD staining (Fig. 3a). However, the increased F-actin staining associated with the actin core is not co-localized with the staining of phosphorylated l-CaD in OCs, since phosphorylated l-CaD became dissociated from the actin core in the RANKL-induced cells with (Fig. 3b) and without l-CaD overexpression (Fig. 1c). In our previous report, using l-CaD decoy peptides mimicking phosphorylation sites at l-CaD, we have shown that l-CaD phosphorylation decreased podosome formation and the substrate-resorbing functions in differentiated OCs with the RANKL-induction [15]. In addition, the same report [15] using phosphorylated (p-l-CaD) and dephosphorylated l-CaD (dp-l-CaD) mutants expressed in cells before and after RANKL inductions also indicated that deposphorylated l-CaD increased, but phosphorylated l-CaD decreased, the RANKL-induced OC differentiation characterized by altering the TRAP-staining activity, cell size, and cell fusion into multinucleated OCs. Apparently, the phosphorylated/deposphorylated l-CaD plays a role in the regulation of RANKL-induced formation of actin ring in differentiated OCs, which may be further involved in their substrate-degrading activities.

In this study, we showed that l-CaD is sufficient and necessary for differentiated OCs to modulate their ability to spread (Fig. 5) and the adhesion force in the cell periphery (Fig. 6). It is likely that l-CaD which regulates the contractile interaction between actin and non-muscle myosin, may also establish a dynamic linkage between adhesion ring and the F-actin core as the podosome formation in differentiated OCs.

Cell-cell fusion to form multinucleated OCs is a critical step in osteoclastogenesis [36]. The strategy used to disturb the downstream effect on the actin cytoskeleton remodelling for controlling OC fusion might be considered as a potential target for therapeutic intervention on the bone-related diseases, including osteoporosis. Previously, a study using various pharmacological agents showed that actin reorganization played a role in the control of cell size of OCs during the cell fusion [4]. Consistently, the present study using the experimental approach with gain- and loss-of-l-CaD expression also showed that l-CaD is involved in the control of the cell size of differentiated OCs in concomitant with the change of cell fusion index (Fig. 4). The l-CaD-mediated actin cytoskeleton remodelling may serve as a key mechanism to modulate the size of differentiated OCs during OC fusion in osteoclastogenesis.

## Conclusions

In summary, our study indicated that l-CaD found in the actin ring modulates the mechanical property of OC precursor cells for the multinucleation of OC fusion in osteoclastogenesis. Our data suggested that l-CaD acts to affect the down-stream actin regulators for controlling the distribution and stability of the podosome and OC functions.

## Additional files

Additional file 1: **Figure S1.** RAW264.7 cells knocked down by si l-CaD showing (a) decreases in l-CaD protein content, (b) decreases in l-CaD mRNA expression, (c) decrease in mRNA for NFATc1, (d) cFos, (e) TRAP, and (f) CTSK. (TIF 630 kb)

Additional file 2: **Figure S2.** RAW264.7 cells overexpressing l-CaD caused increases in (a) protein content of EGFP-l-CaD (b) exogenous human l-CaD before RANKL induction (top) and other osteoclastogenic genes including NFATc1, c-Fos, CTSK, and TRAP after RANKL induction (bottom), (c) the expression of EGFP in transfected cells before and (d) after RANKL induction. (TIF 12629 kb)

## Abbreviations

AFM: Atomic force microscopy
AP-1: Activator protein 1
cNFATc1: cytoplasmic 1
CTSK: Cathepsin K
DMEM: Dulbecco’s modified essential medium
EGFP: Enhanced green fluorescent protein
FBS: Fetal bovine serum
GAPDH: Glyceraldehyde 3-phosphate dehydrogenase
l-CaD: Non-muscle caldesmon
MAPK: Mitogen-activated protein kinase
mCSF: Macrophage colony-stimulating factor
NF-κB: Nuclear factor-κB
NF-T-cells: Nuclear factor of activated T-cells
OCs: Osteoclasts
PBS: Phosphate buffer saline
RANK: Receptor activator of nuclear factor kappa-B
RANKL: Receptor activator of nuclear factor kappa-B ligand
TRAF: Tumor necrosis factor receptor-associated factor
TRAP: Tartrate-resistant acid phosphatase
α-MEM: Minimal essential medium alpha modification

## References

[1] Boyce BF (2013). Advances in the regulation of osteoclasts and osteoclast functions. J Dent Res.

[2] Boyle WJ, Simonet WS, Lacey DL (2003). Osteoclast differentiation and activation. Nature.

[3] Aeschlimann D, Evans BA (2004). The vital osteoclast: how is it regulated?. Cell Death Differ.

[4] Takito J, Otsuka H, Yanagisawa N, Arai H, Shiga M, Inoue M, Nonaka N, Nakamura M (2015). Regulation of osteoclast multinucleation by the actin cytoskeleton signaling network. J Cell Physiol.

[5] Oikawa T, Oyama M, Kozuka-Hata H, Uehara S, Udagawa N, Saya H, Matsuo K (2012). Tks5-dependent formation of circumferential podosomes/invadopodia mediates cell-cell fusion. J Cell Biol.

[6] Jurdic P, Saltel F, Chabadel A, Destaing O (2006). Podosome and sealing zone: specificity of the osteoclast model. Eur J Cell Biol.

[7] Georgess D, Machuca-Gayet I, Blangy A, Jurdic P (2014). Podosome organization drives osteoclast-mediated bone resorption. Cell Adhes Migr.

[8] Vives V, Cres G, Richard C, Busson M, Ferrandez Y, Planson AG, Zeghouf M, Cherfils J, Malaval L, Blangy A (2015). Pharmacological inhibition of Dock5 prevents osteolysis by affecting osteoclast podosome organization while preserving bone formation. Nat Commun.

[9] Vives V, Laurin M, Cres G, Larrousse P, Morichaud Z, Noel D, Côté JF, Blangy A (2011). The Rac1 exchange factor Dock5 is essential for bone resorption by osteoclasts. J Bone Miner Res.

[10] Sobue K, Muramoto Y, Fujita M, Kakiuchi S (1981). Purification of a calmodulin-binding protein from chicken gizzard that interacts with F-actin. Proc Natl Acad Sci U S A.

[11] Humphrey MB, Herrera-Sosa H, Gonzalez G, Lee R, Bryan J (1992). Cloning of cDNAs encoding human caldesmons. Gene.

[12] Gu Z, Kordowska J, Williams GL, Wang CL, Hai CM (2007). Erk1/2 MAPK and caldesmon differentially regulate podosome dynamics in A7r5 vascular smooth muscle cells. Exp Cell Res.

[13] Eves R, Webb BA, Zhou S, Mak AS (2006). Caldesmon is an integral component of podosomes in smooth muscle cells. J Cell Sci.

[14] Tanaka J, Watanabe T, Nakamura N, Sobue K (1993). Morphological and biochemical analyses of contractile proteins (actin, myosin, caldesmon, and tropomyosin) in normal and transformed cells. J Cell Sci.

[15] Liou YM, Chan CL, Huang R, Wang CA (2018). Effect of l-caldesmon on osteoclastogenesis in RANKL-induced RAW264 7 cells. J Cell Physiol.

[16] Oikawa T, Kuroda Y, Matsuo K (2013). Regulation of osteoclasts by membrane-derived lipid mediators. Cell Mol Life Sci.

[17] Novack DV, Faccio R (2011). Osteoclast motility. Putting the brakes on bone resorption. Ageing Res Rev.

[18] Eleniste PP, Bruzzaniti A (2012). Focal adhesion kinases in adhesion structures and disease. J Signal Transduct.

[19] Destaing O, Saltel F, Géminard JC, Jurdic P, Bard F (2003). Podosomes display actin turnover and dynamic self-organization in osteoclasts expressing actin-green fluorescent protein. Mol Biol Cell.

[20] Linder S, Aepfelbacher M (2003). Podosomes: adhesion hot-spots of invasive cells. Trends Cell Biol.

[21] Destaing O, Sanjay A, Itzstein C, Horne WC, Toomre D, De Camilli P, Baron R (2008). The tyrosine kinase activity of c-Src regulates actin dynamics and organization of podosomes in osteoclasts. Mol Biol Cell.

[22] Touaitahuata H, Blangy A, Vives V (2014). Modulation of osteoclast differentiation and bone resorption by rho GTPases. Small GTPases.

[23] Nagai Y, Osawa K, Fukushima H, Tamura Y, Aoki K, Ohya K, Yasuda H, Hikiji H, Takahashi M, Seta Y, Seo S, Kurokawa M, Kato S, Honda H, Nakamura I, Maki K (2013). Jimi E. p130Cas, Crk-associated substrate, plays important roles in osteoclastic bone resorption. J Bone Miner Res.

[24] Gil-Henn H, Destaing O, Sims NA, Aoki K, Alles N, Neff L, Sanjay A, Bruzzaniti A, De Camilli P, Baron R, Schlessinger J (2007). Defective microtubule-dependent podosome organization in osteoclasts leads to increased bone density in Pyk2(−/−) mice. J Cell Biol.

[25] Pasapera AM, Schneider IC, Rericha E, Schlaepfer DD, Waterman CM (2010). Myosin II activity regulates vinculin recruitment to focal adhesions through FAK-mediated paxillin phosphorylation. J Cell Biol.

[26] Ito Y, Teitelbaum SL, Zou W, Zheng Y, Johnson JF, Chappel J, Ross FP, Zhao H (2010). Cdc42 regulates bone modeling and remodeling in mice by modulating RANKL/M-CSF signaling and osteoclast polarization. J Clin Invest.

[27] Rohatgi R, Ma L, Miki H, Lopez M, Kirchhausen T, Takenawa T, Kirschner MW (1999). The interaction between N-WASP and the Arp2/3 complex links Cdc42-dependent signals to actin assembly. Cell.

[28] Chellaiah M, Hruska K (1996). Osteopontin stimulates gelsolin associated phosphoinositide levels and phosphatidylinositol triphosphate-hydroxyl kinase. Mol Biol Cell.

[29] van den Dries K, Meddens MB, de Keijzer S, Shekhar S, Subramaniam V, Figdor CG, Cambi A (2013). Interplay between myosin IIA-mediated contractility and actin network integrity orchestrates podosome composition and oscillations. Nat Commun.

[30] Raab M, Swift J, Dingal PC, Shah P, Shin JW, Discher DE (2012). Crawling from soft to stiff matrix polarizes the cytoskeleton and phosphoregulates myosin-II heavy chain. J Cell Biol.

[31] Tasca A, Astleford K, Lederman A, Jensen ED, Lee BS, Gopalakrishnan R, Mansky KC (2017). Regulation of osteoclast differentiation by myosin X. Sci Rep.

[32] Bähler M (2000). Are class III and class IX myosins motorized signalling molecules?. Biochim Biophys Acta.

[33] McMichael BK, Scherer KF, Franklin NC, Lee BS (2014). The RhoGAP activity of myosin IXB is critical for osteoclast podosome patterning, motility and resorptive capacity. PLoS One.

[34] Jiang Q, Huang R, Cai S, Wang CL (2010). Caldesmon regulates the motility of vascular smooth muscle cells by modulating the actin cytoskeleton stability. J Biomed Sci.

[35] Wang CL (2008). Caldesmon and the regulation of cytoskeletal functions. Adv Exp Med Biol.

[36] Iwasaki R, Ninomiya K, Miyamoto K, Suzuki T, Sato Y, Kawana H, Nakagawa T, Suda T, Miyamoto T (2008). Cell fusion in osteoclasts plays a critical role in controlling bone mass and osteoblastic activity. Biochem Biophys Res Commun.

