# Supplementary figures and images for: L-caldesmon alters cell spreading and adhesion force in RANKL-induced osteoclasts
Source: J Biomed Sci. 2019 Jan 24;26:12. doi: 10.1186/s12929-019-0505-1 (PMC6345023; doi:10.1186/s12929-019-0505-1)

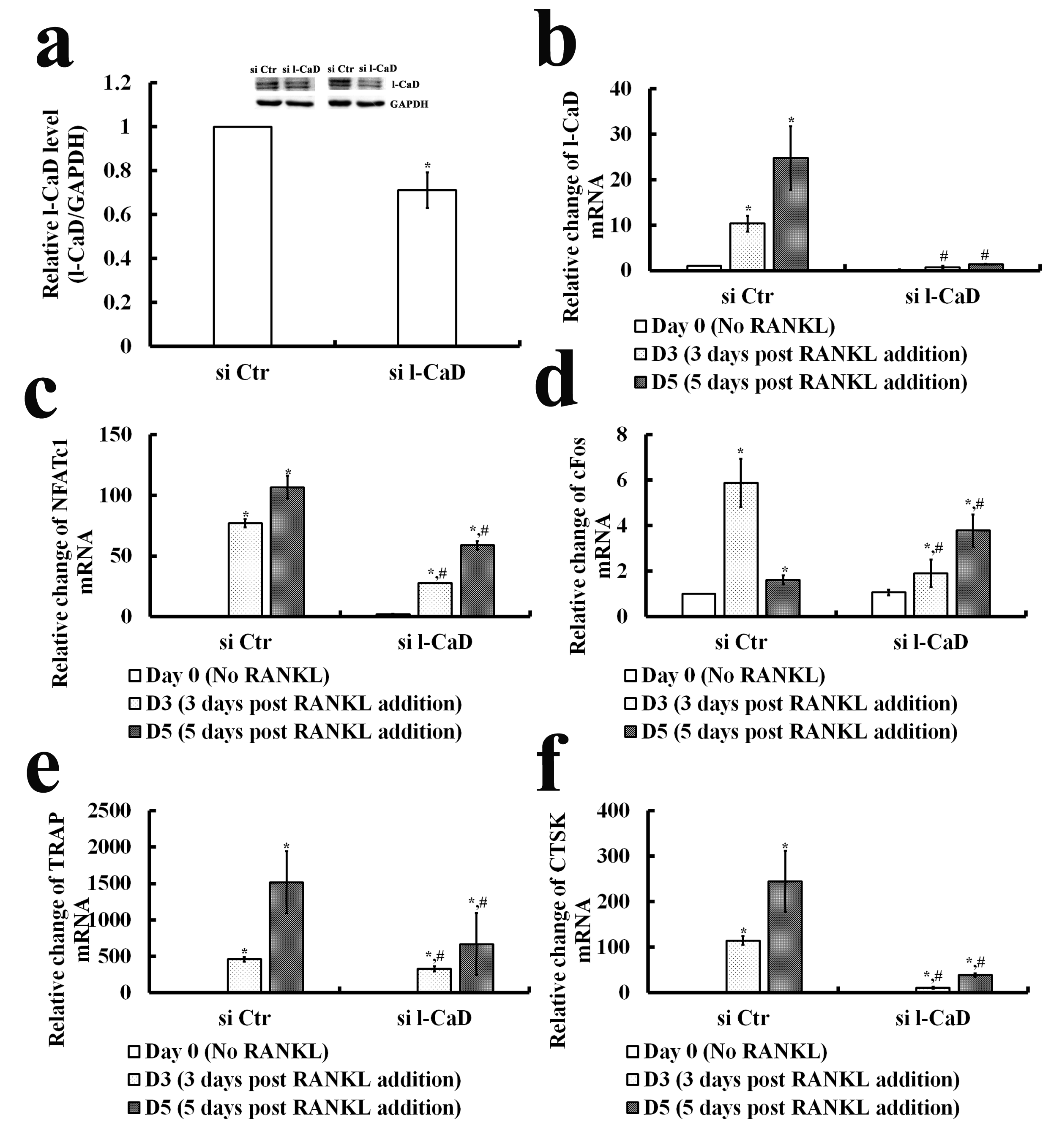

Supplement: Supplementary file 1 — Figure S1. RAW264.7 cells knocked down by si l-CaD showing (a) decreases in l-CaD protein content, (b) decreases in l-CaD mRNA expression, (c) decrease in mRNA for NFATc1, (d) cFos, (e) TRAP, and (f) CTSK. (TIF 630 kb) [file 12929_2019_505_MOESM1_ESM.tif]

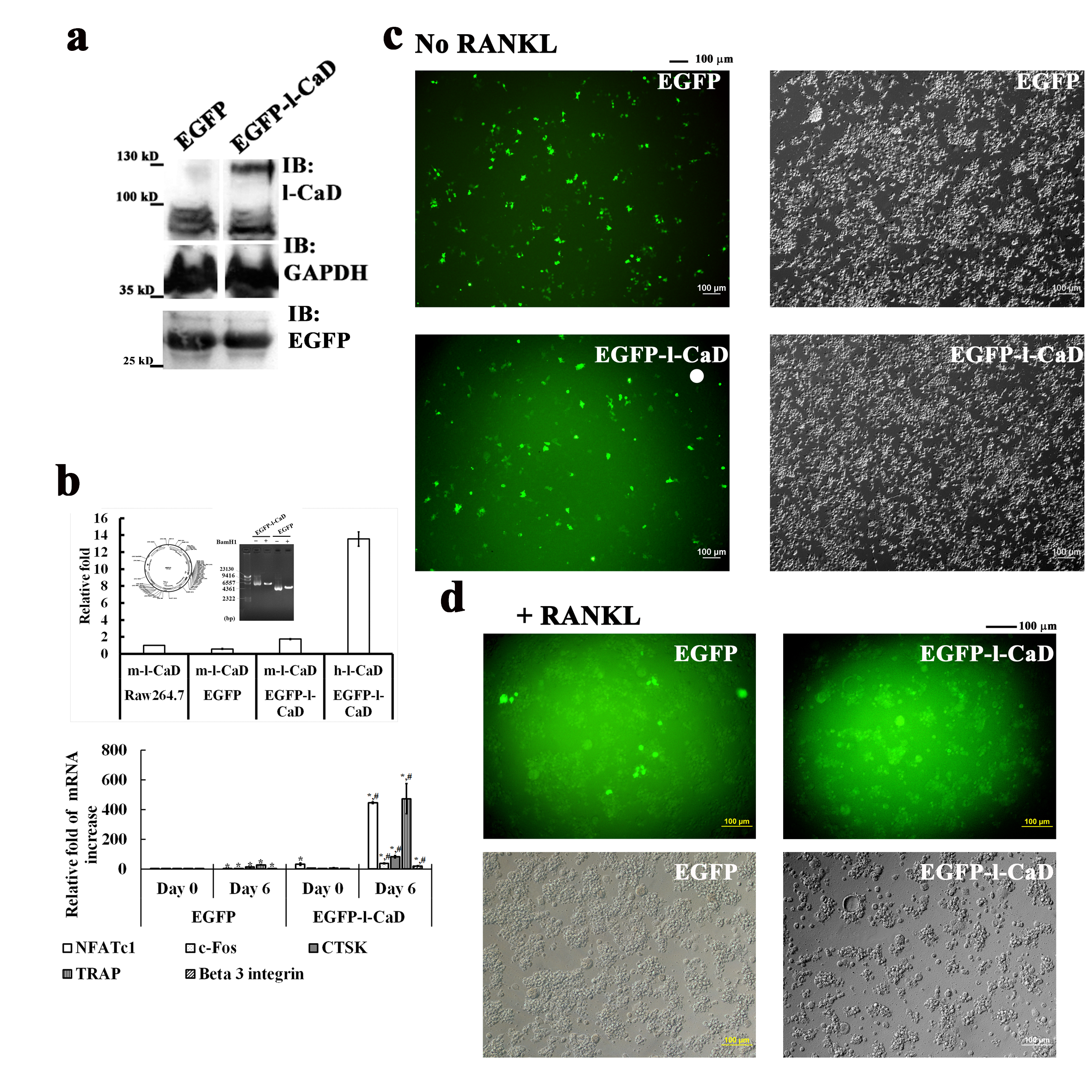

Supplement: Supplementary file 2 — Figure S2. RAW264.7 cells overexpressing l-CaD caused increases in (a) protein content of EGFP-l-CaD (b) exogenous human l-CaD before RANKL induction (top) and other osteoclastogenic genes including NFATc1, c-Fos, CTSK, and TRAP after RANKL induction (bottom), (c) the expression of EGFP in transfected cells before and (d) after RANKL induction. (TIF 12629 kb) [file 12929_2019_505_MOESM2_ESM.tif]
